# Postoperative airway morbidities in pediatric patients

**DOI:** 10.1186/s12871-023-02112-1

**Published:** 2023-06-14

**Authors:** Sun Zhongpeng, Yang Dong

**Affiliations:** Department of Anesthesiology, Plastic Surgery Hospital of CAMS & PUMC, Beijing, China

**Keywords:** Pediatric patients, Airway assessment, Postoperative sore throat

## Abstract

Pediatric airway management is a huge challenge for anaesthetists, and airway-related complications should be actively addressed and focused on.

In a recent prospective observational cohort study, Assefa et al. [1] discovered that endotracheal tube cuff inflation with lidocaine could reduce hemodynamic responses and post-operative airway morbidities in pediatric patients when compared to cuff inflation with air. Their discovery had clinical implications. In addition to the shortcomings mentioned in the article, the authors should address several methodological issues.

First, it was known that pediatric airway management seems to be more challenging for anaesthetists than the adults, so we’d like to know whether preoperative airway assessment was performed in this study and if there was difficult airway. Furthermore, the study did not describe whether video laryngoscope or direct laryngoscope was used, the type of endotracheal tube, the use of stylets, or the number of attempts. Reliable evidence that the number of attempts and the use of various airway management tools were strongly related to postoperative airway complications [2].

Second, The authors did not standardize the extubation protocol, including whether suction was performed before and during extubation, as well as the pressure and frequency of suction, all of which were unknown variables that could potentially and significantly skew their findings. Furthermore, the authors did not classify the patients’ degree of postoperative sore throat, which could easily be assessed using the Prince Henry Pain Scale [3].

Third, the pediatric patients received thiopentone, suxamethonium, isoflurane, vecuronium for anesthetic induction and maintenance, but the authors did not specify which opioids were used for induction and maintenance, as well as the total amount of opioids. There was reliable evidence that using opioids such as sufentanil and remifentanil before and during extubation reduced haemodynamic fluctuation and coughing response [4][5].

Finally, the patients’ satisfaction and postoperative recovery, which were absolutely essential to pediatric prognosis and their families, ought be evaluated using the QoR-15 [6]. We believe that the study’s transparency could be enhanced if the authors provide more pertinent data.

## Data Availability

Not applicable.
